# 

**Published:** 1857-11

**Authors:** 


### CONTENTS OF THE NOVEMBER NUMBER.

### ORIGINAL COMMUNICATIONS.

Page.

ART. I.—Fractures of the Zygoma, or Zygomatic Arch. By Frank H. Hamilton, M, D., - . - 321

ART. II.—Reduction of the Hip-bone by Manipulation; or, Fragments of the Literature of this Method. Compiled by Frank H. Hamilton, M. D., - - - - - - - - - - - 325

ART. III.—Chronic Pleuritis. Cases reported by Addison Niles, M. D., Lockport, N. N., - - 330

ART. IV.—Reports of Cases occurring in the Medical Ward of the Buffalo Hospital. Reported by Dr. A. W. Nichols, - - - - - - - - - - - - - - - - - - 337

ART. V.—Abstract of the Proceedings of the Buffalo Medical Association, - - - - - 346

ART. VI.—Clinical Reports of Diseases, Injuries, Surgical Operations, etc.,etc., at the Buffalo Hospital of the Sisters of Charity. Reported by Dr. B. H. Lemon, - - - - - - - 351

ART. VII.—Encephaloid Disease of the Antrum, &c By Prof. Hamilton. Reported by Benj. H. Lemon, M. D.. - - - - - - - - - - - - - - - - - - - 355

ART. VIII.—Trrnsactions of N. Y, State Med. Soc., New Hampshire Med. Society, and Penn. State Med. Society, - - - - - - - - - - - - - - - - - 356

ART. IX.—Monthly Record of Deaths in the City of Buffalo. By C. L. Dayton, M.D., Health Physician, - - - - - - - - - - - - - - - - - - - 363

### ECLECTIC DEPARTMENT.

On the Lonstitution and Physiology of the Bile, - - - - - - - - - - - - 365

### EDITORIAL DEPARTMENT.

Dalron on the Constitution and Physiology of Bile, - - - - - - - - - - - 381

A Collection of Remarkable Cases in Surgery, - - - - - - - - - - - 381

Lesions of the Epiglottic Cartilage, - - - - - - - - - - - - - - 382

